# Curriculum guide for teaching house officers and faculty: applying procedure codes effectively using chemical denervation as a model

**DOI:** 10.3389/fmed.2024.1359230

**Published:** 2024-09-18

**Authors:** Maryam Berri, Noha Beydoun, Martha Johnson

**Affiliations:** ^1^Department of Physical Medicine and Rehabilitation, University of Michigan, Ann Arbor, MI, United States; ^2^Comprehensive Studies Program, University of Michigan, Ann Arbor, MI, United States

**Keywords:** house officer curriculum, faculty education, billing and coding, medical practice management, procedural billing and coding

## Abstract

**Introduction:**

The healthcare system in the United States relies heavily on physician-and house officer-driven initiation of billing and coding for collection of hospital payments and professional fees. Under the umbrella of practice management is the ever-changing and suboptimally taught concept of procedural billing and coding to house officers and faculty. Clinical providers and practitioners initiate billing and coding for performed services based on the procedural visit encounter, supported by the appropriate documentation. Correct charge capture is dependent on accurately linking CPT codes and J codes, including waste documentation, modifiers, and charge collection. We discuss a perspective regarding a new curricular methodology that teaches learners to apply an algorithmic approach for coding CPT codes, J codes, and modifiers for chemical denervation procedures involving high-cost botulinum toxin. We further recommend the use of visuals with algorithm development for other pertinent procedures that are specific to a department.

**Methods:**

We developed a curriculum that includes algorithmic visuals, pre-and post-test questions, and reflections. It was implemented across various learner types.

**Results:**

This chemical denervation curriculum was well-received and impactful in meeting the objectives of the course. It further expanded a learner’s vision of practice management that can be applied to other procedural examples.

**Discussion:**

The results demonstrate a clear gap in practice management education, with pre-education knowledge on applying appropriate codes being particularly low among resident physicians. Learners found the algorithm we developed especially valuable, as it serves as a practical tool for accurately accounting for all aspects of CPT codes, modifiers, and J-codes. The methodology of the algorithmic approach proved to be innovative for avoiding billing write-offs and loopbacks that were beneficial for the training process. Learners indicated that this approach can be applied to other procedural billing.

## Introduction

In increasingly complex clinical care environments, medical trainees are inadequately prepared for billing and coding procedures. In fact, studies have shown that medical trainees and graduates believe that their training for billing procedures under practice management education was inadequate (1–3). While few studies have included aspects of billing and coding in resident education, they are largely in the fields of pediatrics (4, 5), emergency room (6), and inpatient hospital procedures common in internal medicine (7). There is currently no curriculum that teaches residents appropriate billing and coding for procedures to identify and subsequently bill for botulinum toxin chemical denervation procedures. These procedures are used for spasticity management, cervical dystonia, and migraine treatment, which are common but not limited to departments of neurology and physical medicine and rehabilitation. In fact, according to data published by the Center for Medicare and Medicaid Services, excluding commercial insurance payers, there are approximately 462,082 of these procedure codes utilized in 2022 (8). Yet, curricula that detail billing procedures in medical practice education are still overwhelmingly undertaught and not readily available. Consequently, providers initiate chemical denervation procedural billing codes for both inpatient and outpatient settings, often without an effective feedback loop to correct or optimize the coding. The Accreditation Council for Graduate Medical Education transitioned to “competency based, outcomes-oriented education (9)”. Though this educational approach recognizes practice management, this occurs, however, in the context of a changing billing and coding environment, where optimal billing requires stable attention to compliance with external and internal administrative and regulatory requirements. Some institutions may have a robust reconciliation process for charge capture, and others may not. This results in claim denials, decreased work relative value unit (RVU) compensation, and largely avoidable write-offs. Education is the first line of defense to ensure correct charge capture, which will both increase efficiency in practice management and decrease accidental underbilling. This will inevitably result in less time spent on billing errors and more time spent optimizing patient healthcare. We designed a curriculum that will enable learners to appropriately apply Current Procedural Terminology (CPT®) code(s), modifiers, and J codes to chemical denervation with botulinum toxin injection procedures. While this curriculum targets this specific procedure, its algorithmic model can be adapted to other healthcare settings with unique procedural acumen.

Through a curriculum designed to optimize coding procedures that contribute to avoidable write-offs in billing and coding procedures, our aim is to maximize efficiency in teaching such processes. It includes opportunities for residents, fellows, and faculty to determine when to use codes for specific patient care scenarios and to apply each appropriately without common errors. It will enable learners to effectively assign Common Procedural Terminology (CPT®) codes, J codes for reporting medication, and modifiers for different chemodenervation procedures. Students were presented with four different patient case scenarios with a demonstration of how practitioners should code in each different case scenario, utilizing both images and an originally developed algorithm.

Importantly, this curricular perspective is novel in two ways: first, it addresses the gap in training for billing and coding of chemodenervation procedures, specifically in physical medicine and rehabilitation or neurology practice settings, and second, the innovative development of an algorithm for practitioners to utilize in their approach to appropriately assign CPT codes, J codes, and modifiers outside of chemodenervation procedures specifically, as all procedures utilize formats of these codes. While this curriculum focuses on billing items that contribute to payer denials and avoidable write-offs, our aim is to present methods that can both contribute to improvement in practice management education overall and expand the knowledge so that it can be applied to other procedures. This curriculum also targets learners’ pre-and post-course knowledge as a means of implementing best instructional practices in diverse learning environments.

## Methods

The curriculum was designed to teach learners how to apply correct CPT codes, modifiers, and J codes through an innovative algorithmic model that maximizes billing efficiency in practitioner settings and minimizes payer denials and avoidable write-offs. It was developed by a team consisting of a neurorehabilitation physician experienced in chemodenervation procedures, a Certified Professional Coder from an academic institution, and a university instructor (PhD) with focused experience in best instructional practices in pedagogy and curricular development. The pre-and post-tests (including short responses and reflections) were conducted specifically to target the most common errors in chemodenervation procedures and assess curriculum knowledge outcomes. The team met six times to revise, and the final version was ultimately reviewed and approved for use.

The curriculum was taught both over Zoom® software and in person. Given the COVID-19 pandemic restrictions and newly formed teleconference capabilities nationally, the curriculum was taught first virtually via Zoom over a cohort of three residency classes from 2020 to 2023. The curriculum was taught in person at a national conference to faculty in 2022. Facilitators prepared the slide presentation (Appendix A) that includes review information, the algorithm procedure and chart, patient scenarios, and the pre-and post-tests. Prior to the start of instruction, resident learners were emailed a course pack that included the review information, the algorithm, patient case scenarios, and pre-and post-tests (Appendix B). We also included the algorithm as a separate appendix (Appendix C). For the faculty, this was given at the beginning of the course session in person. The curriculum includes a review of J code (HCPCS) and CPT code definitions, along with which modifiers should be applied in different case scenarios. It includes four patient case scenarios of chemical denervation with botulinum toxins procedures with subsequent demonstrations on how to code and capture charges appropriately. Case studies for Zoom sessions were set up as virtual stations on the screen, allowing learners to move through them and practice the correct application of CPT codes, J codes, and modifiers in these simulations. Case studies for the faculty included a doll to aid in visual and tactile learning in an independent station. Virtual learners had the option of printing the course pack of materials and scanning (and emailing) their responses back or use any other software that enabled them to markup the pdf form directly and return it to the facilitators. Faculty in the in-person course had the opportunity to write down responses on paper and submit them back to the facilitator.

Learners were given a pretest to assess prior knowledge, followed by a posttest at the end of the lesson. Additionally, the posttest incorporated reflection responses on new knowledge. Studies across various domains of medicine have shown that assessing learners’ prior knowledge—in this case through the pretest at the start of training material—can help instructors determine knowledge gaps and better target instruction (10). Furthermore, studies have shown that implementing reflection in medical students’ training is not only a means of self-assessment but also an effective instructional practice in ensuring students can synthesize prior knowledge with their new understandings (11–13). In fact, one study that implemented reflection to measure the efficacy of medical students’ training for teaching notes that “Narratives [produced through reflection] revealed candid self-assessments and detailed descriptions of their experiences and what they valued most from the course (13)”. As such, in the posttest, learners were asked to reflect on the overall course and the ways they might apply their knowledge in practice management to optimize charge capture and billing procedures. Reflection responses were also used to evaluate course effectiveness.

### Implementation

The study was implemented by the revenue physician lead for the Department of Physical Medicine and Rehabilitation.

### Equipment and delivery

To effectively deliver the course material, the following equipment was needed: computer equipment, a slide show presentation, and course packs (either downloaded or printed as needed). For the in-person conference session, dolls were provided for marking practice.

### Activating prior knowledge and pretest session (5 min)

The course began with activating the learners’ prior knowledge. In a brief paragraph for each, learners were asked to respond to three questions that essentially asked what they already knew and what they wanted to gain from the session regarding CPT codes and denervation procedures. Learners were then given three additional content-based questions that targeted content directly covered in the curriculum. These same questions would be repeated in the posttest session, which would not only serve as an assessment tool but also as a measure of the effectiveness of the curriculum.

### Review of CPT codes and modifiers (15 min)

We reviewed chemical denervation CPT code definitions. We also reviewed when to apply modifiers and common mistakes providers make when assigning trunk, neck, and extremity codes. The CPT codes used were: 64612, 64615, 64616, and 64642-7. Review cards were included in the learner course packet and displayed on the screen.

### Patient care scenario simulation (30 min)

After review, learners were introduced to the algorithmic model that physicians can utilize to effectively determine which modifier to use in CPT procedures. They were presented with four different patient case scenarios. The virtual session included stations with visual diagrams to practice the correct application of CPT codes and modifiers in the setting of real-case examples, which included details such as muscles injected, units of botulinum toxin utilized, and wasted. Learners completed stations independently and through real-time participation. The hands-on session for faculty learners included the same materials as the virtual teaching, but the content was printed out and a doll was set up to mark and map out case scenarios (see Appendix D).

### Posttest and reflection

The posttest was administered immediately after the patient scenario simulation. The same knowledge-based questions given in the pretest were asked in the posttest. Learners were then asked to reflect on what they learned and how they would utilize this knowledge on CPT coding procedures in their anticipated practice settings. Learners were asked to write out responses in the form of a brief paragraph.

### Whole group discussion and conclusion

A brief wrap-up with a whole group discussion was then facilitated, and learners shared responses to the posttest and shared reflective responses.

## Results

The results reflect data collected over 26 learners (*n* = 26). The curriculum was administered in an average of 75-min lesson sessions on three different dates. They were administered to house officers in separate ACGME-accredited residency and fellowship programs (11 and 12 learners ranging from PGY2 to PGY5, respectively). It was administered again to three faculty in a national conference. All learners (except 1 faculty member) completed the pretest activating prior knowledge assessment, as well as the posttest and short response reflections. They were sent back to the course instructor.

**Figure 1:**
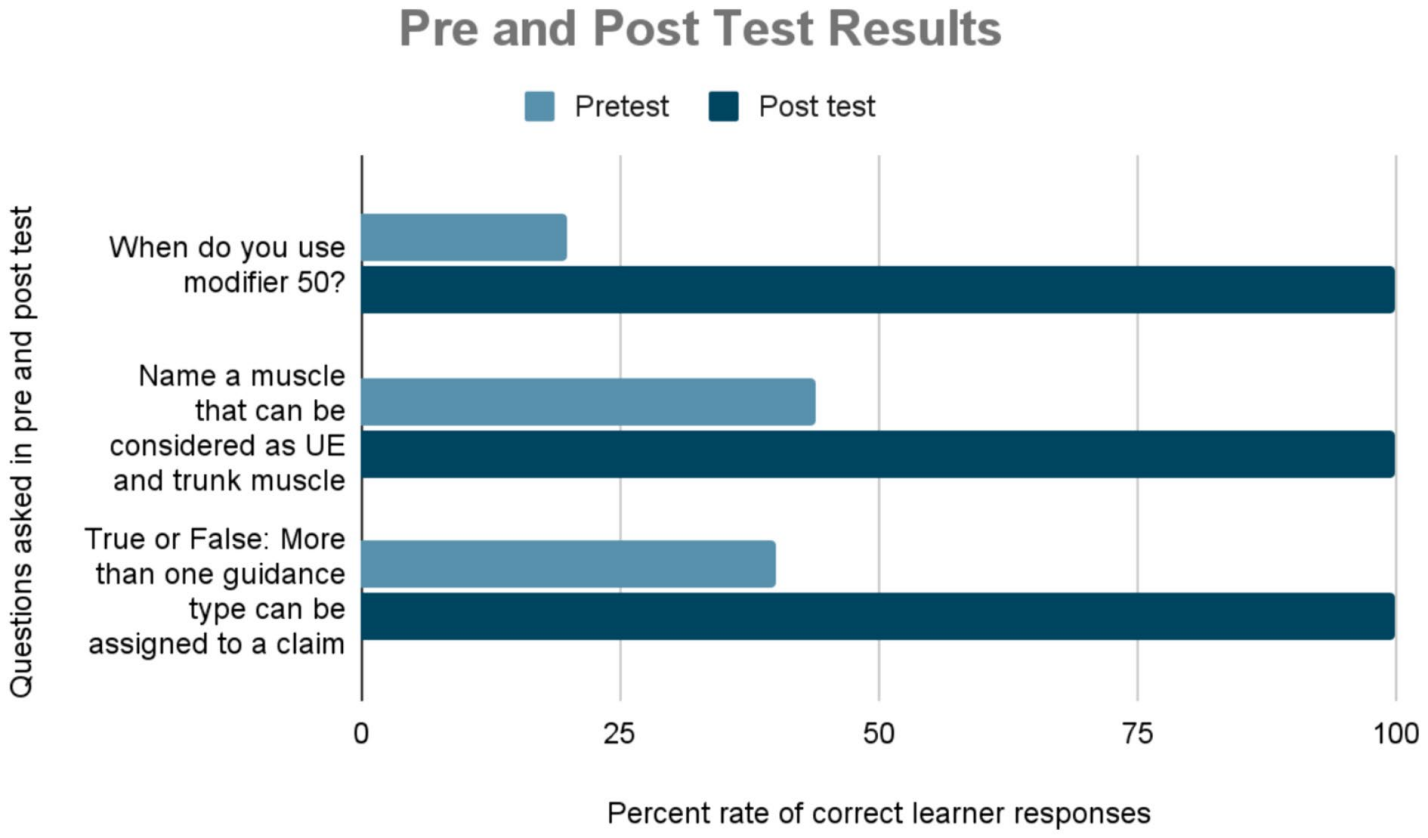
Results of pre-and posttests.

The impact of the curriculum was measured twofold: quantitatively and qualitatively. First, through comparative results of the knowledge-based pre-and post-test questions. That is, learners were asked the same questions in the pre-and post-tests to measure student comprehension and efficacy of curriculum material. Prior to this curriculum, only 20% of students (*n* = 5) knew when to use modifier 50. Additionally, less than half of the class (44%, *N* = 11) could correctly name a muscle that could be considered UE and trunk muscle when coding (*N* = 0 in faculty). Finally, less than half of the class (40%, *N* = 10) knew that more than one guidance type could be assigned to a claim with the use of ultrasound. These percentages changed drastically in the posttest, increasing to 100% after the curriculum was taught, demonstrating efficacy in methodology and curriculum (Figure 1).

**Figure 2:**
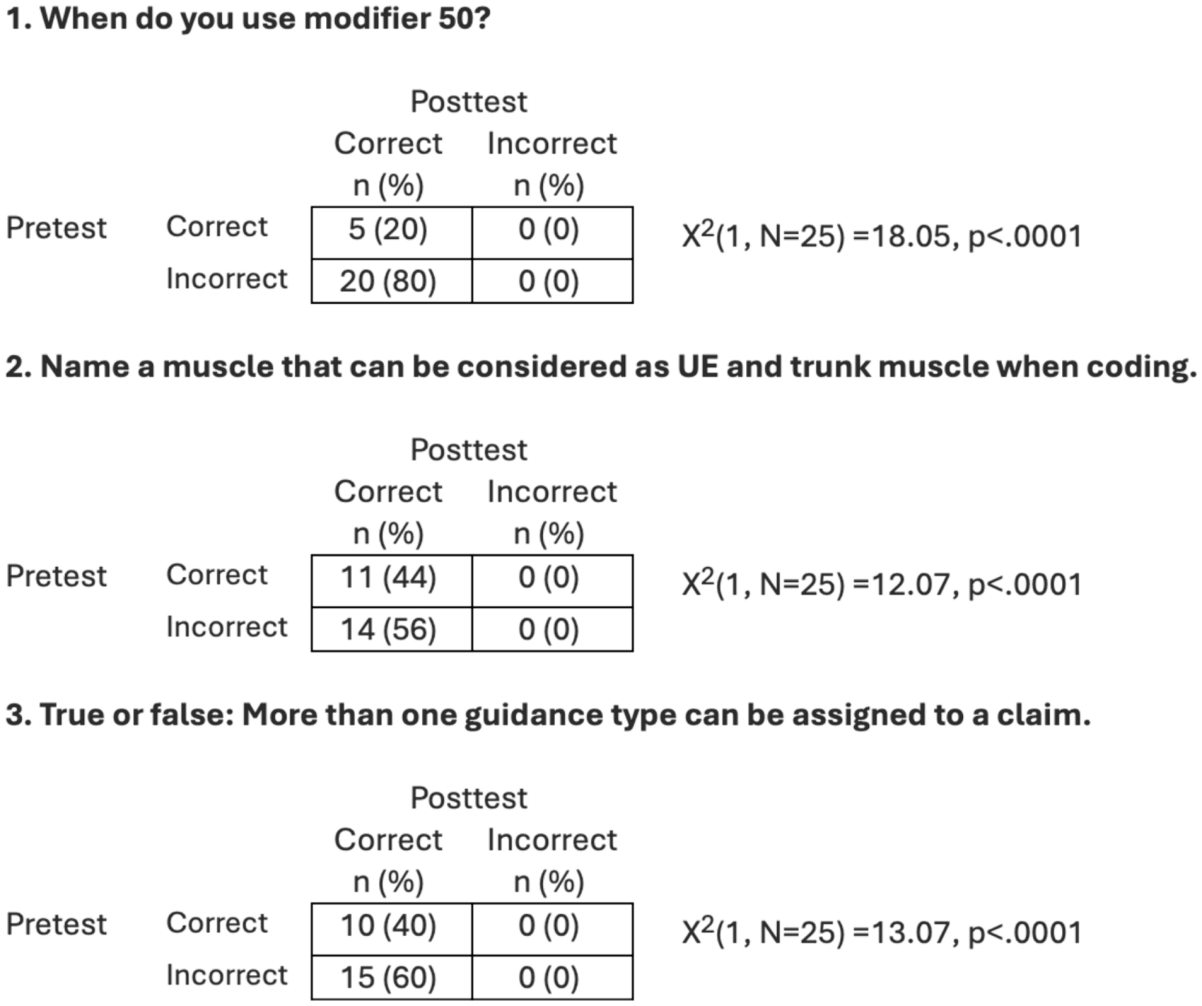
Results of Chi-square analyses with related samples, specifically McNemar’s change tests.

An analysis was conducted to examine whether the proportion of correct responses to the three questions changed from pretest to posttest. Chi-square analyses with related samples, specifically McNemar’s change tests, were conducted. The results are presented in Figure 2. For all three questions, the proportion of correct answers was significantly higher at posttest than pretest.

The impact of the curriculum on learning was also measured qualitatively through pre-and post-test reflection responses. In the pretest, these questions complemented those of the short answer pretest but asked learners to reflect on their current knowledge of chemodenervation procedures. The pretest reflection questions were intended to invite the learner to begin to assess their knowledge base. Responses indicated knowledge gaps and uncertainty among students in various domains of CPT procedures in practice management. Questions with all their reflective responses were typed into a Word document to identify common themes. These questions elicited open-ended answers to learners’ current knowledge of chemical denervation procedures and their codes. The themes included a majority of responses that indicated low to no knowledge and frustrations with the lack of knowledge.

Question 1: What do you know about billing and coding for chemodenervation procedures?

- “I know I may not being coding correctly.”
- “We start doing Botox as a PGY-4. I do not know anything about it.”
- “At this time, my knowledge is limited. However, I understand that it is $1,500 per 100u of Botox. It is prepaid to the administrator.”
- “I know there is a sheet in the clinic with codes. No one truly taught us how to use it but we try to code them based on definition. I do not know if my coding is ever wrong or incorrect because no one cross checks my work or gives me feedback. Its great we are asking this question because I have felt not confident in this area. There are no websites or books to teach us this.”

Question 2: What billing codes would you typically use for Chemical denervation procedures?

- “99214, 99215 are encounter codes-I’m not sure maybe they start with 994… something”
- “I do not know any of the codes.”
- “I do not know what codes are used but I believe there is a procedure modifier code that is added to a visit level code.”
- “I do not know which billing codes are used for denervation. I believe it depends on EMG guidance or lack thereof. It depends on how many muscles are injected as well.”

Question 3: When considering practice management in medicine, what knowledge gaps do you have with CPT codes, J codes, and/or modifiers?

- “I find that there are nuances which can be frustrating especially when learning those on my own. I have not yet used J codes and rarely used modifiers for chemodenervation toxin procedures.”
- “At this time, as a PGY3, I have not been in a situation where I have learned about CPT codes, J Codes, or modifiers.
- “I feel I am slightly exposed to this when in clinic but typically the attending chooses the code and it is not emphasized why later in residency. I feel I currently have significant gaps in coding and billing.”
- “I have heard of CPT codes and modifiers but I am not sure what they mean.”

Accordingly, in Question 1, students expressed clear uncertainty and knowledge gaps in billing and coding. One student indicated that they were unsure of where to go to learn this information. For Question 2, when asked about specific codes, students did not know which set of codes was for denervation procedures. For Question 3, the common theme was, again, overall uncertainty with respect to coding and billing in practice management. One student was frustrated by the knowledge gap and noted that they have to learn this on their own, while another noted they depend on the attending provider to tell them which code, and they are often uncertain why a specific code was chosen. Overall, the pretest indicated significant deficiencies in the billing and coding curriculum.

The posttest reflection questions were intended to invite students to assess their knowledge gained after the course and envision how this might impact their practice moving forward. All responses were typed out into a Word document to obtain informal themes. Learners’ comments indicated that they found both the case studies and the algorithm particularly helpful. Many indicated they would use the algorithm in their future practice. Furthermore, this curriculum substantially increased their understanding of billing aspects of practice management. The reflections indicated intended application in their future practice. The questions and responses included:

Question 1: Which parts of the course were most effective in increasing your understanding of CPT billing procedures?

- “The use of the algorithm along with most, if not all, CPT codes we utilize. The discussion of modifier especially J and JW codes as this was unbeknownst to myself and most others.”
- “The entire course was outstanding. The explanations and reference tables for codes were very helpful but the case examples were very useful critical thinking/application of learned knowledge.”
- “The algorithm was incredibly helpful to use through the cases, and something I plan to use moving forward.”
- “The intro laid a solid foundation for other broad strokes regarding definitions of CPT codes (like office visit), and modifier GC (for teaching physician staffing). Then it moved into specific for chemodenervation but the principles can be applied to other general procedures. The cases included medical knowledge and were interactive regarding muscle pathophysiology and innervation while presenting the billing and coding component in a simple and easy to apply manner. The algorithm was the star of the course!”
- “I thought this presentation was very well put together. Good amount of explanation of the codes at the beginning of the lecture, the algorithm was a helpful flow sheet, and the practice cases helped to bring it together for understanding and application.”

Question 2: In what ways do you think this information will aid you in practice management?

- “I think it will help me advocate appropriate billing for what I know at this time throughout my fellowship and beyond. Knowing this information may help peek interest of practices where I may apply for jobs.”
- “This is my first real exposure to billing and will serve as the framework for my future billing.”
- “It broadened my understanding on practice management. I am more aware of how easy it is to code incorrectly and how important it is to be fluent in applying these codes. The billing and coding generated by a physician is legally and ethically our responsibility.”
- “As a PGY2 with very little experience with CPT codes at this point, I found this very helpful in thinking about codes and the major impact it can/will have on my future practice.”

Question 3: How will you change your approach to appropriately applying CPT to denervation injections? What impact do you think this lesson will have in your approach to billing and coding in practice management overall?

- “I will hopefully take this training into all of my chemodenervation coding in the future.”
- “I now will be placing the appropriate codes for these procedures. The impact might be large enough for our program to consider hiring more faculty which may give us more time for educational endeavors as residents on inpatient rehab. Overall, I think I may be questioning procedural codes moving forward.”
- “This will alter/formulate my style and billing practices for my future. This course will help me daily in my future practice.”
- “Initially, I did not have an approach as I did not have clinical experience to appropriately applying CPT to denervation injections, so this has impacted my practice tremendously.”
- “My new approach will not always include this algorithm and as I place the coding into the EMR I will cross check it against this course material. I will also teach others the correct so we do not continue to make the same mistakes in our department.”
- “I would like make my own flowsheet to use with knee and shoulder injections so I can visualize and not miss anything in charge entry.”

Student reflections in the posttest indicated an overall increase in knowledge and application for denervation procedures and billing and coding. The overall tone from learners was strikingly similar, and there was no seemingly unfavorable reflection/response eliminated from the examples above. For the first question, where learners were asked about the most effective parts of the curriculum, they expressed that the algorithm and case studies in particular were most helpful. For the second question, learners were asked to reflect on application in practice management, to which many expressed that it strengthened their understanding of billing and coding substantially. Finally, the last question asked learners to reflect on the broader implications of the lesson to their practice. Most learners reported an increased knowledge that they believe will alter their billing approach in the future. One learner even noted they would potentially create their own flowsheet (or algorithm) for other parts of the body.

## Discussion

Our results reveal a clear gap in practice management education, with both house officers and faculty demonstrating suboptimal knowledge in applying appropriate procedural charge capture. Most students had expressed little to no exposure to billing and coding well into their residency training, and this extended into faculty responses during the course as well. This curriculum not only addresses the gaps in CPT coding education for chemodenervation procedures but also invites learners to think about the importance of billing and coding in their future and current practices as it applies to other procedures.

**Figure 3:**
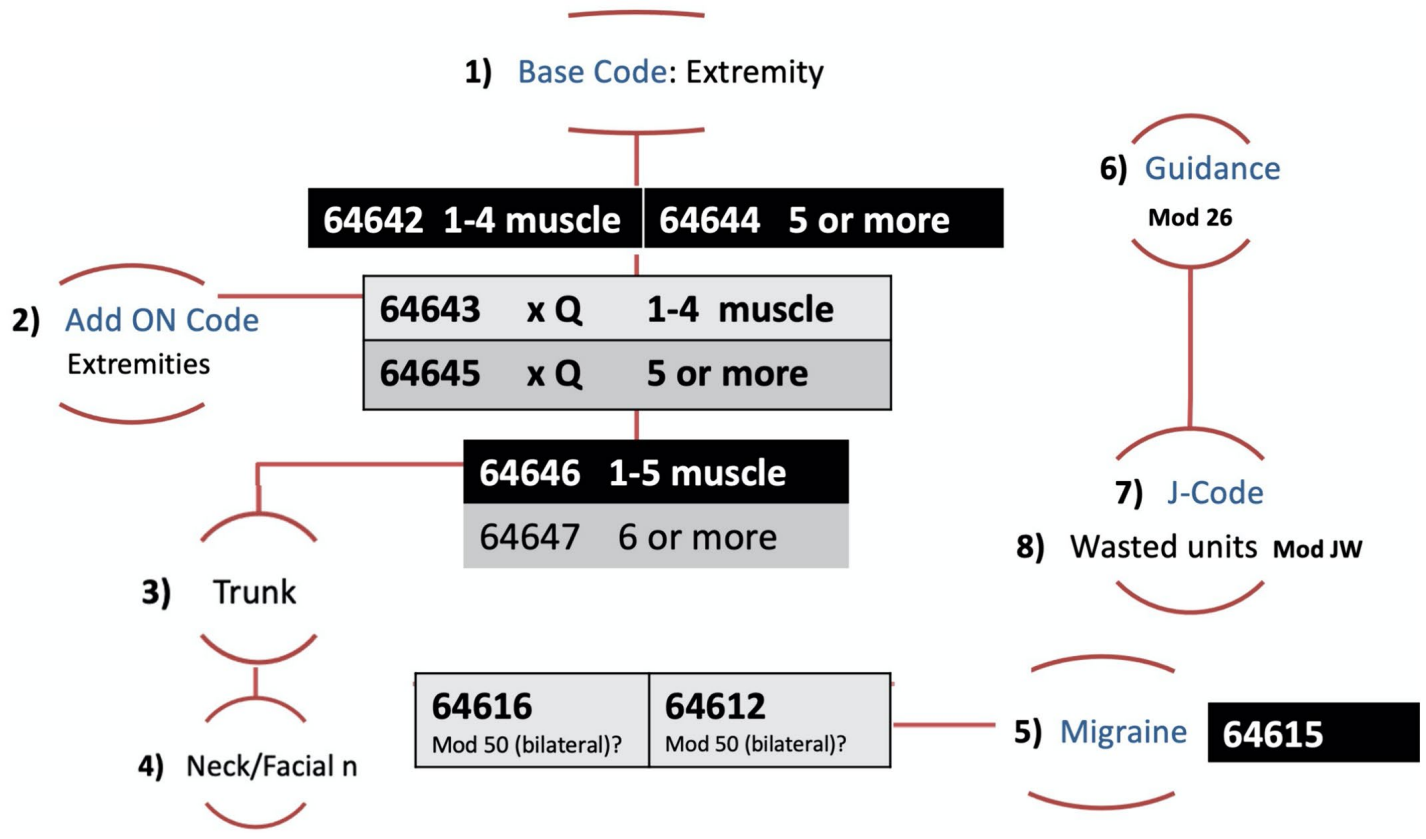
Algorithm for chemical denervation procedural charge capture.

The curriculum and algorithm (Figure 3) stressed the importance of capturing all units of neurotoxin injected, including wasted units, to ensure accurate reimbursement for high-cost neurotoxins. It also maximized billing RVUs by applying the appropriate modifiers. Future considerations on this topic include completing a scoping review to understand the lay of the land in the overall setting of billing and coding education, procedure, or otherwise, in order to understand the starting points for these major gaps.

Our evaluation method for the course relied heavily on student reflection rather than quantitative surveys, which hardly encouraged practitioners to think critically. By implementing reflection, learners were given opportunities to self-assess their prior knowledge and revisit what they had learned. Importantly, they drew connections between pre-and post-course content knowledge and envisioned how this might impact their practice moving forward—to which all learners indicated specific ways they would implement the course, including using the algorithm and case examples with visuals.

Learners indicated that the algorithm, which we developed as a tool practitioners can utilize to accurately assign CPT codes, modifies, and J codes, was especially valuable. The algorithm proved to be an innovative approach for avoiding billing write-offs and loopbacks. It was beneficial for training and, as learners indicated, also applicable to other procedures.

A major limitation of this study is that it was conducted on a smaller sample of students, ranging from PG2 to PG4 residents. Notably, the faculty course was held during a COVID-19 spike as well, with fewer attendees than expected. Nevertheless, the consistent results that showed substantial knowledge growth in every student suggest that it could be adapted effectively in larger sections when applicable.

Another limitation is the timing of the posttest assessments. Since the posttest was administered nearly immediately after the patient care scenario, we acknowledge the question of long-term knowledge retention. That is, the posttest timing likely assessed recall rather than practical application in different contexts. Nevertheless, the results do indicate increased knowledge, while the reflections envisioned where such implementation might occur in their own future practice.

In addition, future sessions could include a review of Medicare’s coverage policy [LCD-local coverage determination-L33458] and other payer guidelines, which include indications, limitations, and medical necessity for coverage. If a procedure is performed without meeting the payer’s criteria, charges may be denied and ultimately may result in write-offs, another major contributor.

We envision that this curriculum can continue to be easily adapted to in-person settings with the case scenarios set up with models/or dolls to increase visual and tactile learning as restrictions continue to lessen. We also envision that this approach to teaching and developing an algorithm with visual case studies that includes all components of procedural billing needs (J codes, modifiers, and reporting waste) can be applied to other practice management teaching activities in the future.

## Data Availability

The original contributions presented in the study are included in the article/Supplementary material, further inquiries can be directed to the corresponding author.
