# Supplementary material for: Curriculum guide for teaching house officers and faculty: applying procedure codes effectively using chemical denervation as a model
Source: Front Med (Lausanne). 2024 Sep 18;11:1359230. doi: 10.3389/fmed.2024.1359230 (PMC11444958; doi:10.3389/fmed.2024.1359230)
Supplement: Supplementary file 1 [file Data_Sheet_1.PDF]

What do you know about billing and coding for Chemodenervation toxin procedures?

What billing codes would you typically use for denervation procedures.

When considering practice management in medicine what knowledge gaps do you have with CPT codes, J codes, modifiers?

Pre- test

- When do you use Modifier 50?_____________
- Name a muscle that can be considered as UE and trunk muscle when coding. _______________
- True or false: More than one guidance type can be assigned to a claim ______________

CPT description w/ wRVU	Modifier 50	Modifier 26
**64642** PC- Chemodenervation of ONE extremity: 1-4 muscle(s) RVW 1.65	**n/a**	yes
**64643** **PC- Chemodenervation of ONE extremity, each additional extremity: 1-4 muscle(s) RVW 1.22**	**n/a**	yes
**64644** PC- Chemodenervation of ONE extremity: 5 or more muscle(s) RVW 1.82	**n/a**	yes
**64645** **PC- Chemodenervation of ONE extremity: each additional extremity 5 or more muscle(s) RVW 1.65**	**n/a**	yes
**HIGHLIGHTED are BASE CODES**		

CPT description w/ wRVU	Modifier 50	Modifier 26
**64612** PC- Chemodenervation of facial nerve unilateral RVW 1.41	yes	yes
**64615** PC- Chemodenervation of facial/trigeminal/cervical muscle migraine **(Bilateral)** RVW 1.85	no	yes
**64616** PC- Chemodenervation of neck muscle(s)/excluding larynx **(Unilateral)** RVU 1.53	yes	yes

Guidance: A Professional Charge	Modifier
Visual/palpation only. No guidance charge	n/a
**95873** PC Electric Stimulation Guidance for Chemodenervation	26
**95874** PC Needle EMG Guidance for Chemodenervation	26
**76942** PC Ultrasound Guidance for Chemical Denervation	26

Trunk muscle (s) Chemodenervation		
**64646** PC Chemodenervation Trunk muscle(s):1-5 1.8 RVW		
**64647** PC Chemodenervation Trunk muscle(s):6 or more 2.11 RVW		

1) Base Code: Extremity

64642 1-4 muscle	64644 5 or more

2) Add ON Code

Extremities

64643	x Q	1-4 muscle
64645	x Q	5 or more

64646	1-5 muscle
64647	6 or more

3) Trunk

4) Neck/Facial n

64616<br>Mod 50 (bilateral)?	64612<br>Mod 50 (bilateral)?

5) Migraine 64615

6) Guidance

Mod 26

7) J-Code

8) Wasted units Mod JW

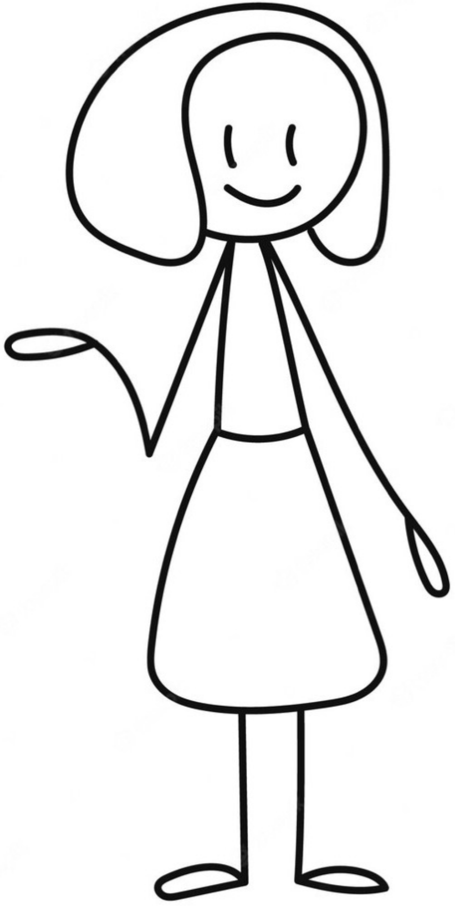

Case 1:

100 units of IncobotulinumToxinA was diluted using 4 cc of preservative free saline. E-stim was used to localize muscles. 50 units were injected into right adductor magnus (50U), (25U) in the Adductor Longus and (50U) in the left adductor Magnus.

How do we code this? Please place markings on figure for where injected.

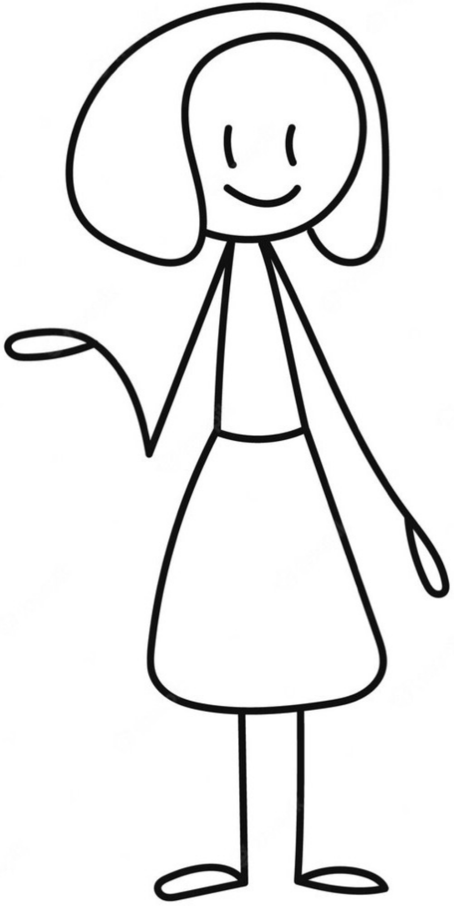

Base Code:
Add On Code:
Trunk
Neck/ facial n
Migraine
Guidance
J code:
Wasted units

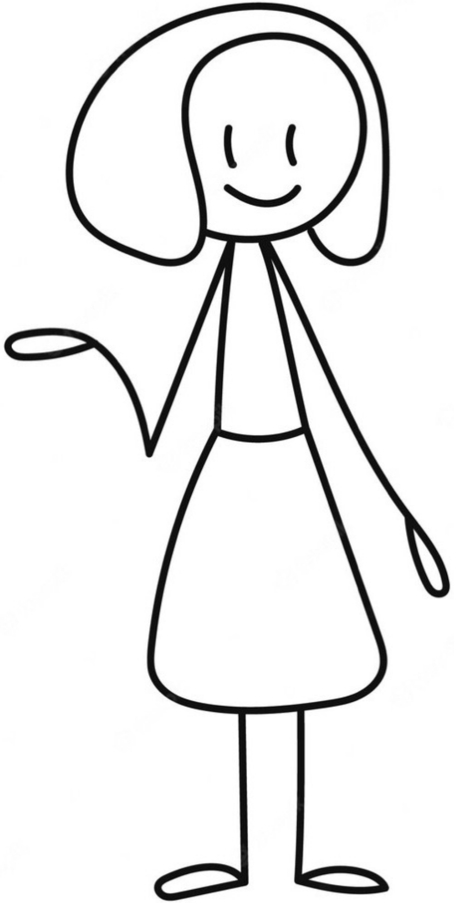

Case 2: 200 units of OnaBotuliumtoxinA was prepared with dilution with 4 cc Normal Saline. Lower extremity chemodenervation is unchanged. **Please trace screen for markings on these muscles. How do we code this**?

Corrugator	5	5
Procerus	5	
Frontalis	10 (2 sites)	10 (2 sites)
Temporalis	20 (4 sites)	20 (4 sites)
Occipitalis	15 (3 sites)	15 (3 sites)
Trapezius	15 (3 sites)	15 (3 sites)
Cervical paraspinal	10 (2 sites)	10 (2 sites)

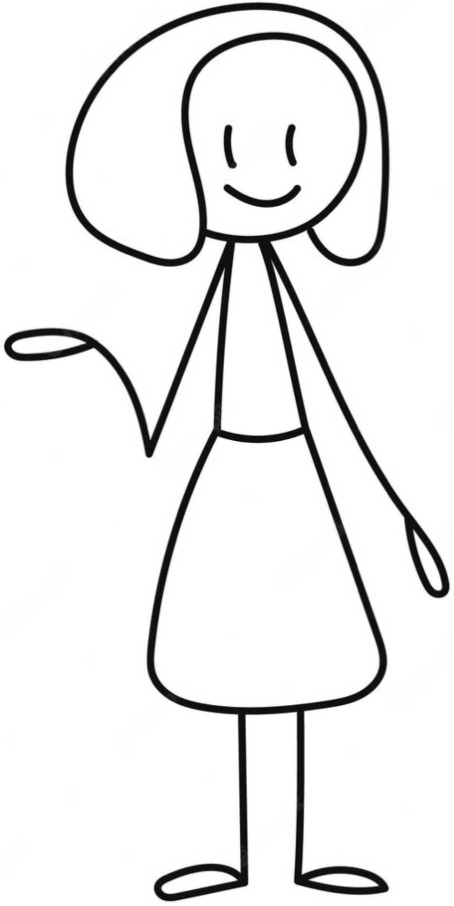

Base Code:
Add On Code:
Trunk
Neck/ facial n
Migraine
Guidance
J code:
Wasted units

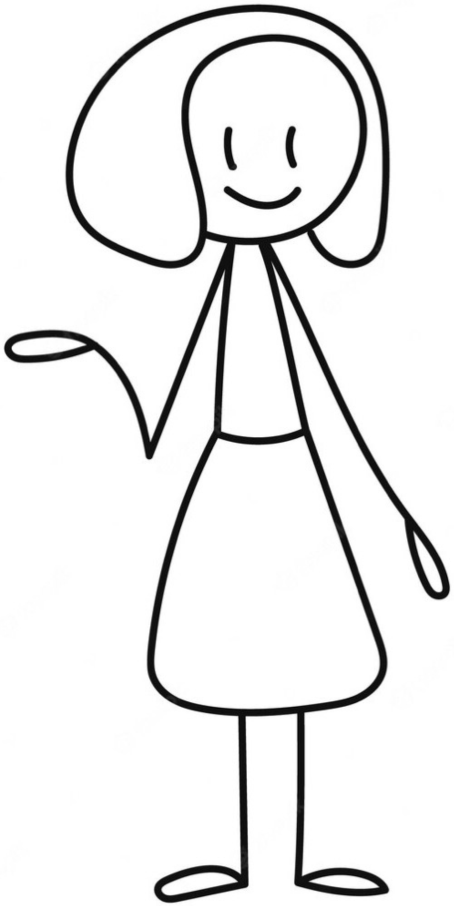

Case 3

Amanda suffers a left MCA stroke with right hemiparesis with pain due to tone.

*E-Stim guidance for her <u>Upper extremity:</u>
Pronator Teres (20U) FDP (20U), FDS (20U), brachialis(20U), FPL(10).

*US guidance for her <u>Lower Extremity:</u>
Gastrocnemius medial and lateral head (25U each), soleus (25U), posterior tibialis (25U) as well as her baseline adductor tone bilaterally (50U).
You dilute 300 units with 6cc preservative free saline of Onabotulinumtoxin A.

Place markings where injected. How would you code this procedure?

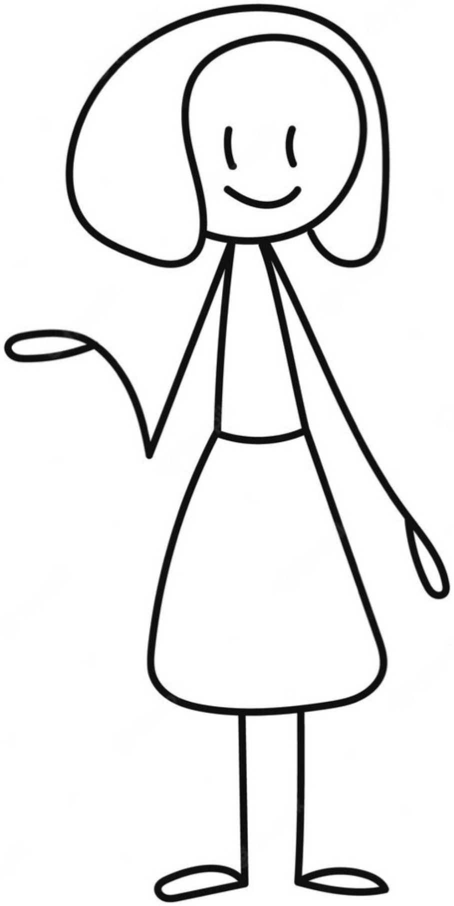

Base Code:	
Add On Code:	
Trunk	
Neck/ facial n	
Migraine	
Guidance	
J code:	
Wasted units	

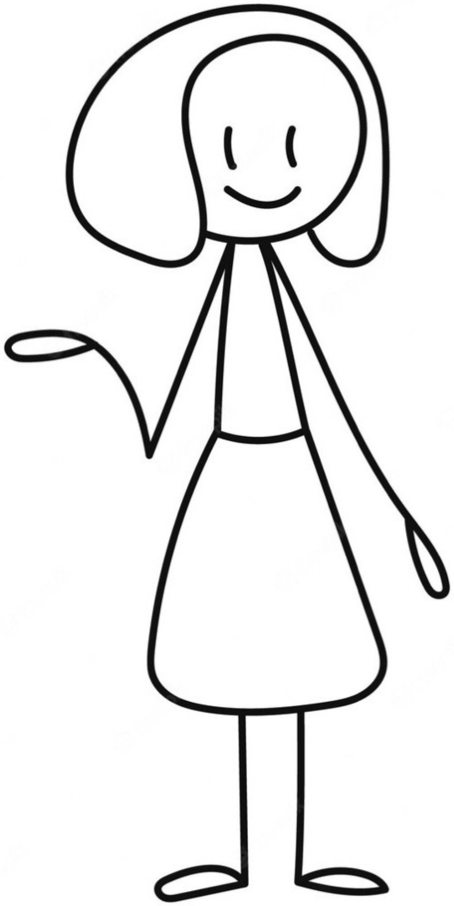

Case 4: Lura is a patient with C3 ASIA A Vent dependent SCI with TBI that is noted to have her head constantly turned to the right with her chin flexed into her body with shoulder adduction. This position causes pain and skin irritation underneath the chin and on the chest with a stage 2 pressure injury.

You elect to use EMG for guidance to inject her bilateral SCM (left 30 units, right 20 units, bilateral scalenes (15 units) and left pectoralis m (20 units) with Onabotulinumtoxin A. You dilute 100 units with 4cc preservative free saline.

Please place markings where injected. How would you code this?

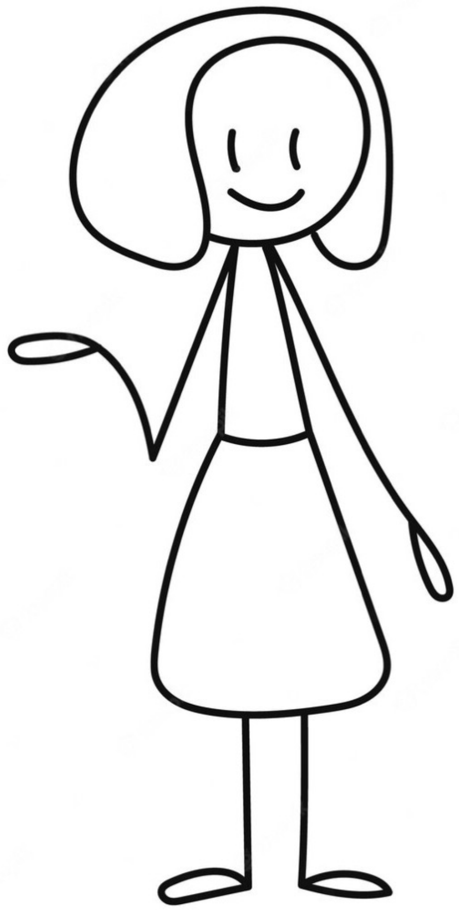

Base Code:
Add On Code:
Trunk
Neck/ facial n
Migraine
Guidance
J code:
Wasted units

Post test

- When do you use Modifier 50?_____________
- Name a muscle that can be considered as UE and trunk. ________________
- True or false: More than one guidance type can be assigned to a claim _______________

Which parts of the course were most effective in increasing your understanding of CPT billing procedures?

In what ways do you think this information will aid you in practice management?

How will you change your approach to appropriately applying CPT to denervation injections? What impact do you think this lesson will have in your approach to billing and coding in practice management overall?
