# Supplementary figures and images for: Curriculum guide for teaching house officers and faculty: applying procedure codes effectively using chemical denervation as a model
Source: Front Med (Lausanne). 2024 Sep 18;11:1359230. doi: 10.3389/fmed.2024.1359230 (PMC11444958; doi:10.3389/fmed.2024.1359230)

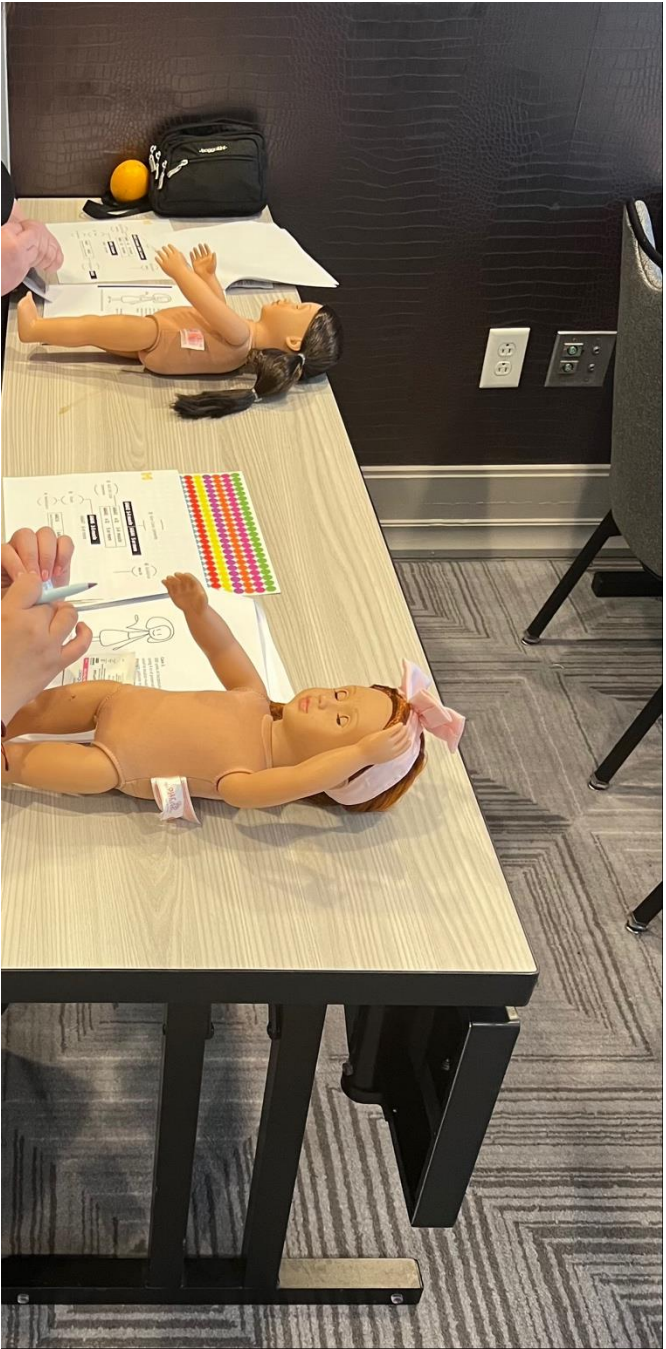

Supplement: Supplementary file 2 [file Data_Sheet_2.PDF]
